# Fire ants perpetually rebuild sinking towers

**DOI:** 10.1098/rsos.170475

**Published:** 2017-07-12

**Authors:** Sulisay Phonekeo, Nathan Mlot, Daria Monaenkova, David L. Hu, Craig Tovey

**Affiliations:** 1 School of Mechanical Engineering, Georgia Institute of Technology, Atlanta, GA 30332, USA; 2 School of Physics, Georgia Institute of Technology, Atlanta, GA 30332, USA; 3 School of Biology, Georgia Institute of Technology, Atlanta, GA 30332, USA; 4 Industrial and Systems Engineering, Georgia Institute of Technology, Atlanta, GA 30332, USA

**Keywords:** emergent, swarm, bivouac, self-assembly

## Abstract

In the aftermath of a flood, fire ants, *Solenopsis invicta*, cluster into temporary encampments. The encampments can contain hundreds of thousands of ants and reach over 30 ants high. How do ants build such tall structures without being crushed? In this combined experimental and theoretical study, we investigate the shape and rate of construction of ant towers around a central support. The towers are bell shaped, consistent with towers of constant strength such as the Eiffel tower, where each element bears an equal load. However, unlike the Eiffel tower, the ant tower is built through a process of trial and error, whereby failed portions avalanche until the final shape emerges. High-speed and novel X-ray videography reveal that the tower constantly sinks and is rebuilt, reminiscent of large multicellular systems such as human skin. We combine the behavioural rules that produce rafts on water with measurements of adhesion and attachment strength to model the rate of growth of the tower. The model correctly predicts that the growth rate decreases as the support diameter increases. This work may inspire the design of synthetic swarms capable of building in vertical layers.

## 1. Introduction

Aggregations of animals such as birds, fish and social insects have attracted much interest because of their ability to accomplish complex tasks efficiently without a clearly designated leader, and despite the severely limited information and cognitive processing ability possessed by each individual [1–5]. Termites, for example, build mounds that can reach 8 m in height, more than 70 000 times their body length [6,7]. The taller the structure, the more torque it experiences from wind and gravity, and the more unstable it becomes. Since termites do not seem able as individuals to perceive the mound size, it is poorly understood how they maintain its physical stability as it grows. In addition, the ability to build such tall structures has no parallel in the world of robotics. While robots numbering in the hundreds have been used to build horizontal structures [8], and flying robots have been used to build scaffolding [9], roboticists still cannot build structures like skyscrapers using decentralized independent units. In this study, we focus on tower building by fire ants.

Fire ants *Solenopsis invicta* reside in the Patanal wetlands of Brazil. In response to the rainy seasons, they evolved the ability to link their bodies together to build rafts to keep their colonies together. They use sticky pads at the ends of their feet to attach to surfaces [10–12]. These sticky pads also enable them to link to each other to build a three-dimensional network [13,14], whose material properties are sensitive to applied forces. Tests using rheometers, sensitive devices to measure a material’s resistance to deformation, show that ants are shear-thinning like paint. At applied forces of around 2 ant weights, ants release their grip on each other and begin to flow like a fluid [15]. Ants only maintain their grip if they experience stresses lower than this threshold. Consistent with these measurements, we have observed that ants build rafts that are only 2.5 ants tall. Rafts that are originally taller than this height flatten within minutes [13,16–18]. This inability to stably support high stresses makes fire ants unlikely tower builders.

**Figure 1.**
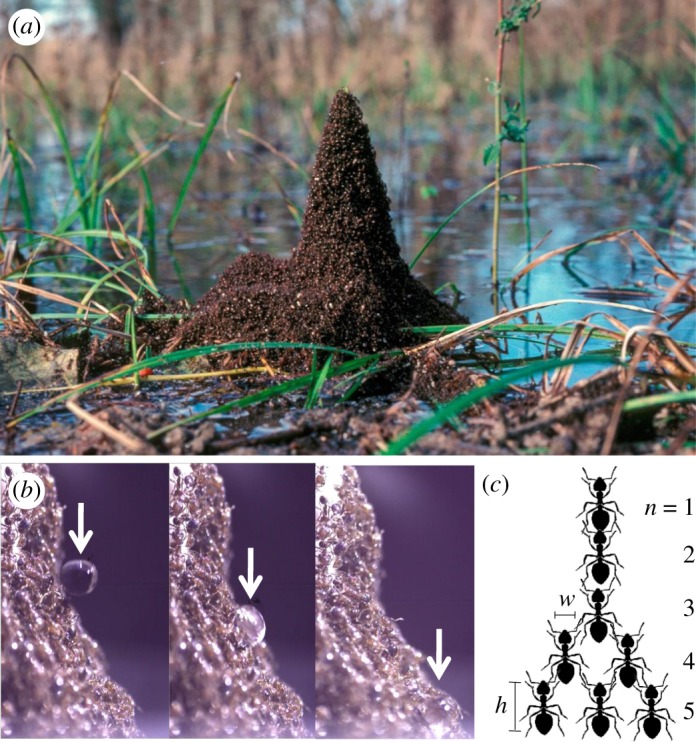
The shape of an ant tower. (*a*) A trumpet-shaped ant tower built around emerging vegetation in the Atchafalaya Basin Swamp in Louisiana. Photo courtesy of CC Lockwood. (*b*) Water droplet rolling down an ant tower. (*c*) Schematic of five layers of an ant tower with carrying capacity *α*=2 ants.

Floating fire ant rafts anchor themselves to vegetation, and in the process, build tower-like structures such as the one in figure 1*a*, photographed in Atchafalaya Swamp, Louisiana. An ant tower such as this one serves as shelter from rain and water currents. It also serves as a base of operations before ants can build the underground tunnels where they will ultimately reside. Little is known about fire ant tower construction and shape. More is known about analogous structures, the overnight bivouacs of army ants [19]. Such structures are built in the crevices of tree trunks and other vegetation, and can be over 30 ants deep.

In this study, we investigate tower building by fire ants. We begin in §2 with reporting our experimental methods. In §3, we report our theoretical models for predicting tower shape and growth rate. We present our hypotheses and experimental results in §4 and compare our results to theoretical predictions. In §5, we discuss how well our predictions support our hypotheses. In §6, we discuss the implications of our work and avenues for future research.

## 2. Experimental methods

In §2.1, we present our experimental methods for caring for ants, inducing them to build towers in the laboratory and measuring their material properties. In §2.2, we present models for tower shape and building rate.

### 2.1. Ant husbandry

We procure fire ant (*S. invicta*) colonies from roadsides near Atlanta, GA. Colony selection aims for an average ant weight of 1 mg. We remove colonies from the soil and place them into bins according to methods by Chen [20]. Ants are fed baby food and pet food three to four times a week, and we perform experiments using nine different colonies with regular replenishment of their water supply.

### 2.2. Tower building

We collect 10 g of ants in a beaker and then swirl them to produce a ball of ants. This ball is placed in the centre of a 150×150×15 mm clear acrylic box. To induce them to build a tower, we place a central support within the box. A Teflon rod is dusted with talc powder and lowered into the centre of the sea of ants filling the box. If the rod is not coated with Teflon, the ants adhere to it so easily that they climb without support and do not form a tower. We prevent ants from escaping over the sides of the box by coating the box with Fluon and talc powder on the sides. Within a few minutes, ants begin to form a tower centred around the Teflon rod (see the electronic supplementary material, movie S1). We film the process from the side using a high-definition Sony HDR-HC9 video camera. After the tower reaches equilibrium, we return the ants to their bins for at least 2 h before sampling again from the colony, which generally contains between 20 and 40 g of ants. We use the same colony at most twice per day. We also run experiments using 15 and 20 g of ants, confirming qualitatively that greater numbers of ants indeed build similar towers. Room temperature stays between 23^°^C and 25^°^C.

To measure the time for an ant to fill a slot on the rod, we closely film the building of towers on rods of two diameters, 4 and 9.5 mm. We limit our count to slots whose vacancy time can be viewed without obstruction by ants. For each diameter, we track 51 empty slots from when they became available until an ant fills the slot and remains on top of the ant below it. This requires frame-by-frame observation across approximately 250 min of video. Electronic supplementary material, video S2 shows a typical video from which we collect the data. Ants tend to fill slots from the bottom of the tower upward. The diagram in figure 5*a* shows that once ants attach to the rod, the slots above them become available. Since videos are filmed from the side, only 8–10 slots can be viewed at a time, with the rod obscuring the remaining slots.

### 2.3. Ant strength measurement

To measure the amount of weight an ant can carry, we apply weights to it. We place a 0.05 g transparency sheet atop a single ant and gradually increase the number of sheets until the ant does not walk. Since we can observe the ants through the transparent sheets, we can easily tell when the ants stop walking in any direction. After we record the weight at which walking ceases, we increase the weight further until the ant does not move any of its limbs. After we record this second weight, we remove the sheets and observe the ant for visual signs of injury, and we observe the ant’s movements for changes in behaviour.

Ants have two types of connections in the tower, an ant–ant connection, and an ant–Teflon connection. We measure the strength of an ant’s leg-to-leg connection by tying two ants, of the same colony, each to an elastic string. We place the two ants in contact to stimulate a leg-to-leg connection. Once the ants grip each other’s legs, we pull the elastic string, which causes it to stretch. We then use Hooke’s Law to measure the force.

We measure the force with which an ant grips Teflon by placing Teflon rods of diameters 4.7 mm (*N*=9), 12.8 mm (*N*=10) and 14.5 mm (*N*=6) on a Metler Toledo analytical balance. We place one tethered ant on the Teflon rod and slowly pull the ant upwards until it releases its hold. We record the whole process using a Sony HDR-HC9 video camera and use Tracker to find the maximum amount by which the measured weight decreases.

### 2.4. X-rays of towers

To observe ant movement within the tower, we dope 5 g of fire ants with a radiographic contrast medium (GE Omnipaque Ioxehol solution) by mixing it with their drinking water and allowing the ants to drink for at least 2 days before building a tower. Immediately before tower building begins, we mix 5 g (5000 ants) of doped ants thoroughly with 5 g (5000 ants) of undoped ants from the same colony. In total, we use the same number of ants as our other experiments. We place the ants in a 100 mm diameter×55 mm tall container with an 8 mm diameter Teflon rod located in the centre of the dish. We allow the ants to build a tower with the process captured using an imaging system that includes the following: Spelman XRB502 Monoblock X-Ray source and Amorphous Silicon Digital X-Ray Detector PaxScan 2020+ (Varian Medical Systems). We operate the X-ray at a current of 2.5 mA and a voltage of 100 keV. In the resulting images, ants that have ingested a lot of the contrast medium show as dark spots for which we use ‘Tracker’, a free product of Open Source Physics. This program allows us to trace individual paths (dark spots) of ants frame-by-frame throughout the sinking process. Approximately 2% of the doped ants ingest enough radioactive iodine to be visible. Owing to the limited aperture of the X-ray machine, only a fraction of those ants show up in the images. The doped ants sometimes get in each other’s way or disappear from view of the X-ray. Therefore, we only tracked ants that we could observe for at least 10 continuous frames. If an ant were stationary, it would show up as a stationary dark spot. We measure the sinking speed from data points taken at different heights and different distances from the rod.

## 3. Mathematical modelling

Here, we present models of the shape of the tower and its rate of growth. In our past work on raft construction, we found ants followed three rules, which yielded accurate predictions for raft growth rate [17,18]. These rules are as follows:

1. Do not move if ants are on top of you.
2. If atop other ants, repeatedly move a short distance in a random direction.
3. Upon reaching available space adjacent to non-moving ants, stop and link with them.
4. The top layer of the tower is not stable unless there is a complete innermost ring of ants gripping each other around the rod.

We include the fourth rule based on our observations, which we report in the Results section. We assume these rules hold true for the following two models.

### 3.1. Tower shape

To model the shape of the ant tower, we idealize the tower as consisting of horizontal layers of ants, as shown in figure 1*c*. In reality, the layers are not perfectly defined. Ants that are horizontally adjacent are not necessarily at exactly the same height. However, they maintain their relative positions once they are connected and do not interpenetrate each other. Designate layer 1 as the top layer, and layer *n*+1 as being immediately below layer *n*. Suppose one ant has a carrying capacity of *α* ants. Ants are known to be strong, so *α* will be larger than 1. Let *X*_*n*_ be the number of ants in layer *n*. The ants in layer *n*+1 must be able to support the ants in layers 1 through *n*. Therefore,

3.1 $$\alpha X_{n+1}\geq \sum_{i=1}^{n}X_{i}.$$

At maximum load, the above equation will hold with equality. Hence at maximum load, it is a good approximation to write

3.2 $$\alpha X_{n+1}=\sum_{i=1}^{n}X_{i}=X_{n}+\sum_{i=1}^{n-1}X_{i}=X_{n}+\alpha X_{n}=X_{n}(\alpha +1).$$

According to the above equation, the number of ants in a layer grows as a geometric sequence with ratio 1+1/*α*. However, if the ants in the second layer supported the top layer at maximum load, *X*_2_<*X*_1_. That is, there would be fewer ants in layer 2 than in layer 1. Instead, we observe that layers 2 and 3 are the same size as layer 1. We therefore refine our formula as follows by setting the top few layers to be the same size.

Let *β*=*X*_1_ be the number of ants in the top layer.

3.3 $$X_{n}=\left\{ \begin{array}{ll} \beta & \text{if}\;1\leq n\leq \alpha \\ \beta {\left(1+\frac{1}{\alpha }\right)}^{n-\alpha } & \text{if}\;n>\alpha . \end{array}\right.$$

The hypothesis for this model, equation (3.1), requires that there be enough horizontal area beneath yet close to layer *n* for *X*_*n*+1_ ants to fit. As the slope approaches the horizontal, this requirement becomes impossible to satisfy. Instead, the tower would have to be buttressed by additional ants. We did not observe such a phenomenon, nor did we expect to do so since a simple estimate indicates that a much larger number of ants would be needed to evoke it.

### 3.2. Tower growth rate

The inset in figure 5*a* depicts the discretization of the top of the tower into a ring of spaces. Given Rule 2, the time to fill an empty space on a ring should be independent of the fill time of other spaces, and have a memoryless distribution. That is, if after *t* seconds a space has not been filled, its remaining expected time to fill is the same as it was at zero seconds. In terms of the random variable *Y* equal to the time to fill a space, *E*[*Y* | *Y* ≥*t*]=*E*[*Y* ] for all *t*≥0. The exponential distribution [21] is the unique memoryless continuous distribution on the non-negative real numbers, with cumulative distribution function (CDF)

3.4 $$P(Y\leq t)=1-e^{-t/\lambda },\;\text{where}\;t\geq 0,$$

and λ is the mean time to fill an empty slot. Defining *w* to be the width of an average ant, let *S*≈*πD*/*w* be the total number of spaces around a rod with diameter *D*. When *S* spaces are empty, the time *Z* until *S*−1 spaces are empty is

3.5 $$Z=\min_{1\leq j\leq S}(Y_{j}),$$

where the *Y*_*j*_ are jointly independent exponentially distributed variables, each with mean λ. Therefore, the time *Z* to fill the first space is distributed so that

3.6 $$P(Z\geq t)=P\left(\overset{S}{\underset{j}{⋂}}Y_{j}\geq t\right)=\prod_{j=1}^{S}P(Y_{j}\geq t)={\left(e^{-t/\lambda }\right)}^{S}=e^{-t/(\lambda /S)}.$$

Hence, *Z* has exponential distribution with mean λ/*S*. Once one space is filled, by the memoryless property, the remaining time until all the spaces are filled is the same as if we start with *S*−1 empty spaces, which is equal to λ/(*S*−1), plus the time as if we start with *S*−2 empty spaces. Inductively, the expected time *T* to fill all *S* empty spaces is

3.7 $$T=\sum_{j=1}^{S}\frac{\lambda }{S-j+1}=\lambda \left(1+\frac{1}{2}+\frac{1}{3}+\cdots +\frac{1}{S}\right)\approx \lambda \log S.$$

### 3.3. Mathematical estimate of the tower’s surface area

We derive an estimate for the ratio of the number of ants on the tower surface to the number of ants that would have to be added to the tower to make it one ant-height higher. The estimate is needed to explain why ring formation limits the tower height growth rate, despite the exponentially large number of ants in the tower as a function of height.

Since the ant orientations vary greatly, we treat individual ants as balls or cubes of unit radius. All measurements are in terms of these units. Let *N* be the height of the tower, *r*= radius of the rod, *f*(*n*)= annular radius of level *n* of the tower, where *N*, *r* and *f* all have units in numbers of ants. For convenience define *ζ*=1+1/*α*.

No explanation is needed for the first several tower layers, where the size of the ring is comparable to the number of ants needed for an additional layer. Therefore, we assume that the number of ants in the bottom tower layer greatly exceeds the ring circumference, that is, *ζ*^*N*^≫2*πr*. The constant *β* factors out of the computations, so without loss of generality, we set *β*=1.

We first estimate *f*(*n*). According to the shape model, and by Taylor series expansion on *r*,

$$\zeta^{n}=\pi ({\left(f(n)\right)}^{2}-r^{2})$$

and

$$f(n)=\sqrt{r^{2}+\frac{1}{\pi }\zeta^{n}}\approx \sqrt{\frac{1}{\pi }\zeta^{n}}+\frac{r^{2}}{2\sqrt{1/\pi \zeta^{n}}}.$$

Then,

$$\frac{df(n)}{dn}\approx \frac{\log\zeta }{2\sqrt{\pi }}\zeta^{n/2}-\frac{r^{2}\sqrt{\pi }\log\zeta }{4\zeta^{n/2}}.$$

Consider a horizontal slice of the tower with thickness *dn*. To first order, its exposed surface area is

$$\begin{array}{rl} dS & =2\pi (f(n)+r)\sqrt{dn^{2}+df^{2}}\\ & \approx 2\pi \left(\frac{1}{\sqrt{\pi }}\zeta^{n/2}+r\left(1+\frac{r\sqrt{\pi }}{2\zeta^{n/2}}\right)\right)\sqrt{1+{\left(\frac{df}{dn}\right)}^{2}}\;dn\\ & \approx 2\pi \left(\frac{1}{\sqrt{\pi }}\zeta^{n/2}+r\right)\sqrt{1+{\left(\frac{df}{dn}\right)}^{2}}\;dn\\ & \approx 2\pi \left(\frac{1}{\sqrt{\pi }}\zeta^{n/2}+r\right)\frac{\log\zeta }{2\sqrt{\pi }}\zeta^{n/2}\;dn\\ & =\zeta^{n}\log\zeta \left(1+\frac{r}{\zeta^{n/2}}\sqrt{\pi }\right)dn\\ & \approx \zeta^{n}\log\zeta \;dn, \end{array}$$

where the second approximation is accurate except for small values of *n*, which contribute little to the total surface area of the tower, and the third and fourth approximations use *ζ*^*N*^≫2*πr*>1. The total surface area of the tower is therefore estimated as

$$S=\log\zeta \int_{1}^{N}\zeta^{n}\;dn\approx \zeta^{N}.$$

For the estimated value *α*=3, there are *ζ*^*N*−*α*^=*ζ*^*N*^/*ζ*^3^ ants in the bottom layer. The estimated ratio of ants on the tower surface to the number of ants that would have to be added to make it one ant-height higher is therefore

$$\zeta^{3}={\left(1+\frac{1}{3}\right)}^{3}\approx 2.4.$$

## 4. Results

### 4.1. Tower shape

**Figure 2.**
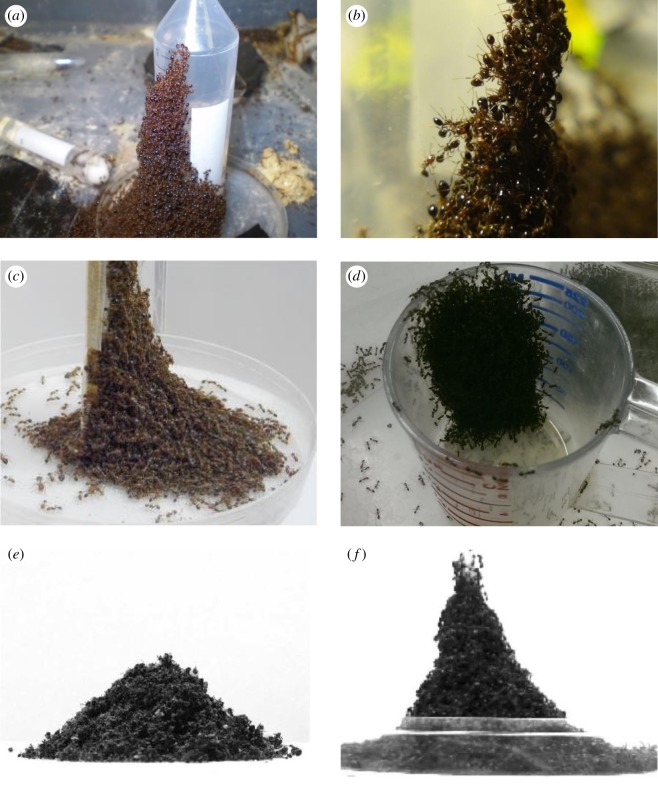
Spontaneous tower building in the laboratory. (*a*) Fire ants build towers around water sources. (*b*) Close-up of the ants within the tower. In both images, a vertical test tube dispenses water into a Petri dish. Fire ants also build towers for escaping a container. (*c*) Fire ants building a tower on a rectangular block of wood coated with Fluon, liquid Teflon, to render it difficult to climb. (*d*) An ant tower built from within a beaker permits ants to escape the beaker. (*e*) A pile of dead ants has a conical shape. The slope is constant at an angle of repose approximately 36^°^ from horizontal. (*f*) By contrast, the ant tower shows a bell shape.

When we house ants in the laboratory, we observe towers naturally built in the ant’s containment bins, as shown in figure 2*a*–*d*. Ants apparently prefer to build along vertical supports such as walls, rods or upturned tubes that provide water. Sometimes, the towers are bell shaped, but results are variable because of the differing wall friction on each of the vertical supports. To reduce the influence of wall friction, we design experiments where fire ants build towers on slippery rods. To challenge the ants, we use rods varying from 4.7 to 16.0 mm.

We film ants building 26 towers with heights ranging from 7 to 30 mm and an average height of 15.8±5.3 mm. Ants complete their tower building in 25±8 min. The towers are much wider at the bottom than at the top. Moreover, the tower slope is not constant as in a cone. Rather, the slope becomes more horizontal towards the bottom. We observe another benefit of tower building: the tower repels water droplets falling at velocities at least up to 3 *m* s^−1^, the maximum we can attain in the laboratory, equal to one-third of a natural raindrop’s velocity [22]. Rather than penetrating the tower, the drop simply rolls down its surface, as shown in figure 1*b*.

Our preliminary observation is purely qualitative, that the towers greatly widen from top to bottom. Why are the towers not of constant width, as are most skyscrapers? An ant at the base of such a tower 30 ants high would bear the weight of 29 other ants. It is plausible that an ant could not sustain such a load. We hypothesize that for some *α*, ants will not bear loads that exceed the weight of *α* ants. We observe that ants constantly clamber over one another. An ant with one ant atop it initially ends up with more ants atop. On the basis of this observation, we further hypothesize that the preponderance of ants will each bear that maximum weight. Ants at the top few layers of a tower obviously cannot bear the maximum. This hypothesis is analogous to the ‘Towers of constant strength’ proposed by Timoshenko in 1930 [23] and used decades earlier in the design of the Eiffel tower. We employed this hypothesis to predict the tower shape, which is bell shaped, as given in equation (3.3).

The hypothesized shape model makes several testable predictions. First, for a fixed number of ants, the shape should be the same in repeated trials. Second, for any *k*, the top *k* mm of the tower should have the same shape, regardless of the total number of ants. This is because an individual behaviour of bearing a load of *α* ants should not be affected by the population size. That is, an ant *k* mm from the top should not behave differently when above 4 or 14 additional mm of ants. Third, there should exist an *α*>1 such that, for multiple trials with different numbers of ants, the tower shape is consistent with the shape formula given. The principal experiment consists of repeated trials with different numbers of ants and rod diameters. For each trial, we measure the tower width at each level from top to bottom. We check for regularity in shape and determine the best single value of *α* that fits all of the data.

**Figure 3.**
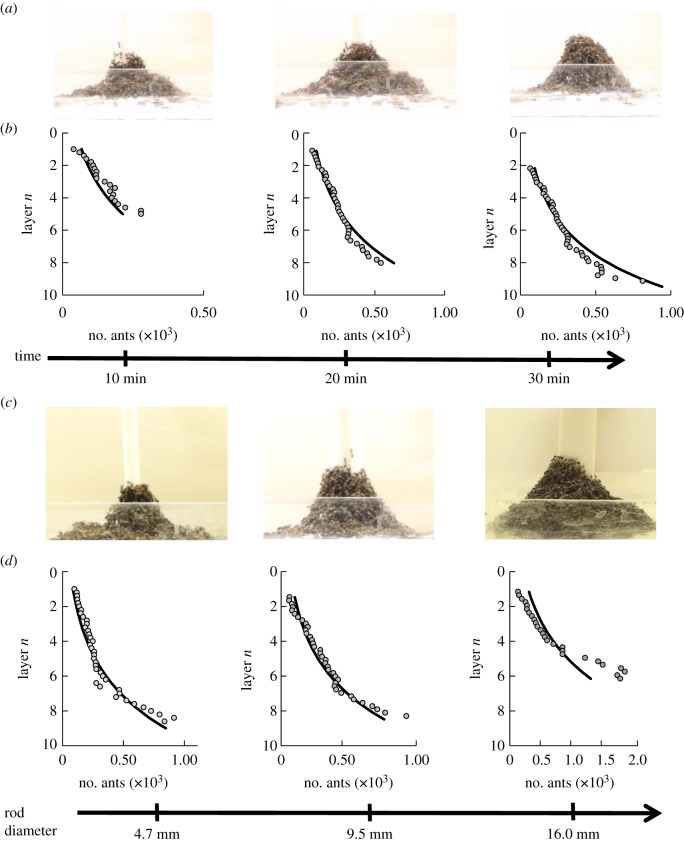
Ant tower profile. (*a*) A time sequence of an ant tower built on an 8 mm diameter Teflon rod. (*b*) The fit of the shape model to the profile of a 10 min, 20 min and equilibrium ant tower. (*c*) The equilibrium shape of ant towers built on 4.7, 9.5 and 16.0 mm rod diameters. (*d*) The fit of the shape model to the profile of the equilibrium ant towers on increasing rod diameters. The circles represent the profiles of the towers. We fit these profiles to the shape model, shown by the solid lines, using carrying capacity *α*=3.0.

In our analysis, we disregard towers less than three layers in height. We were left with 23 videos of ant towers built on rods with diameters ranging from 4 to 16 mm. Figure 3*a* shows the 10 min, 20 min and equilibrium shapes of the towers built around an 8 mm diameter rod. In figure 3*b*, the circles indicate the profile of the ant towers and the line is the model, equation (3.3). The fit is good, as shown by the *R*^2^-values of 0.74, 0.85 and 0.88, respectively (*N*=3). In the model, there are two parameters used. One is the number *β* for number of ants in the top layer. The second is the ant strength *α*. The location of the top layer was visually ambiguous so we used the clearer *β* value of the first two layers for each ant tower. We applied this method to all the ant towers to find the best single fit of *α*=3.0 ants. The value *α*=3.0 is strikingly consistent with the value of 2 ant body weights found in the rheometer tests [15] described in the Introduction. The shape model with this *α*-value was accurate with *R*^2^=0.78 (*N*=23). Figure 3*c* shows the towers built on increasing central rod diameters and figure 3*d* compares the tower profiles with the theoretical predictions. We conclude that the model accurately predicts the overall shape of the tower for a range of rod diameters.

**Figure 4.**
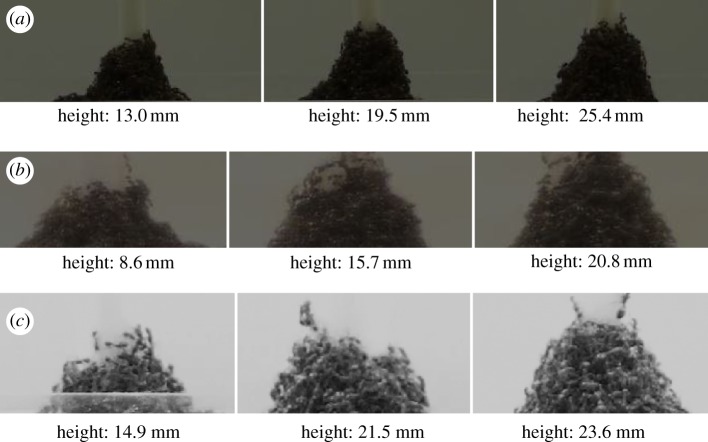
Shape of the top of the tower. We observe qualitatively that the top of the fire ant tower has a fairly consistent shape throughout; the top of the towers built around (*a*) 8, (*b*) 9.5 and (*c*) 11.2 mm diameter rods looks similar regardless of the height of the tower. Qualitatively, we show the close-up view of three trials at varying heights. This shows that the shape at the top of the tower is not affected by the height of the tower.

The procedure for choosing *β* is needed because of the variability in the first few layers in terms of number of ants per layer. Figure 4 shows the variability of the top of the towers for the same diameter at three different times. Our model could not predict this number because these ants were not supporting their maximum load. As a consequence, the fits of the model to the ant towers were not as good at the top of the towers when compared with the bottom.

To determine the biological significance of the ant strength *α*, we performed the compression test on three different ants with an average weight of 1.1±0.15 *mg*. A weight of 88±13 *mg*, or about 83±12 times body weight, prevented the ant from walking. A weight greater than 0.8 g, or about 750 times body weight, prevented ants from moving any limbs at all but did not kill or appear to injure them after we removed the transparency sheets from the ants. These values of 83 and 750, compared with the value *α*=3.0 experienced by ants in towers, are strong evidence that ants stay still within their towers out of their own volition, and not because of their physical limitations.

It is noteworthy that the bell shape is only indicative of live ants. Non-living granular materials such as dead ants will form conical structures with a constant angle of repose, as shown in figure 2*e*. We first euthanize ants with liquid nitrogen and then pour them through a funnel into a Petri dish. The angle of repose, the slope of the pile relative to the horizontal, was 36^°^, very similar to the known conical shape with constant 34^°^ angle of repose for sand piles [24]. In comparison, the tower of live ants has a rapidly decreasing slope relative to the horizontal, traversing downwards, seen in figure 2*f*. We conclude that ants are physically exerting themselves when in the tower rather than merely piling on top of each other.

### 4.2. Tower growth rate

**Figure 5.**
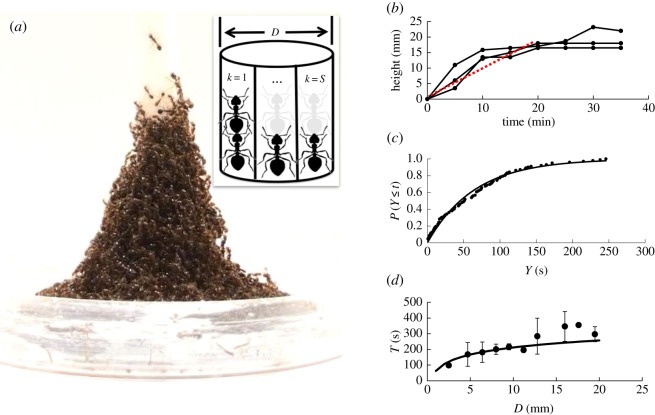
Building rate of ant towers. (*a*) A side view of an ant tower. The inset shows a ring of spaces from *k*=1 to *k*=*S* around a rod of diameter *D* that ants can fill. (*b*) Time sequence of tower height (*N*=3, rod diameter=9 mm). The red dashed line is the average growth rate. (c) The solid circles shows empirical cumulative distribution function (CDF) of the time *Y* to fill a slot, found from video tracking. The solid line is the CDF from the predicted exponential distribution. (*d*) Relationship between ring filling time and rod diameter. The circles represent the experimental time to complete a ring (*N*=3 for each diameter for which an error bar is shown). The line is the predicted time to fill a ring.

 Figure 5*b* shows the time course of the height of the tower. Tower construction is completed at 20 min, when the tower increases to a height of 15 mm, or 5 ant body heights. Roughly, this gives an average rate of growth of the tower of a new layer of ants every 4 min. What sets this rate of growth?

We divide the circumference into vertical ‘slots’ because ants tend to attach around the rod vertically, as shown in inset in figure 5*a*. The number of slots *S*=*πD*/*w* where *D* is the rod diameter and *w* is the ant width of 1 mm. For an 8 mm diameter rod, there are approximately 25 slots available per level. Given that the individual ant movements are random, the time to fill an empty space on a ring should be independent of the fill time of other spaces, and have a memoryless distribution. That is, if after *t* seconds a space has not been filled, its remaining expected time to fill is the same as it was at zero seconds. In terms of the random variable *Y* equal to the time to fill a space, the probability that *Y* is less than some time *t* is given by *P*(*Y* ≤*t*)=1−*e*^−*t*/λ^, where λ is the mean time to fill an empty space. Details are shown in §3.

 Figure 5*c* compares the cumulative distribution of the exponential distribution with empirical data from 102 measurements. We obtained the 102 data points by measuring the time it takes one ant to fill a slot and stay on top of another ant starting from when it becomes available. We performed experiments using central rods of 4 and 9.5 mm in diameter and measured 51 data points for each rod diameter. Visually, the fit is excellent. The Kolmogorov–Smirnov test value is confirmatory with confidence 0.95 (details are given on pages 342–343 of Feller’s book [21]). This gives strong supporting evidence that our hypotheses given in the Mathematical modelling section are correct. Based on the estimated value λ=65 s and ant-height *h*=3.0 mm, the tower growth rate would be $h/\lambda =2.8\;\mathrm{mm}\;\min^{-1}$ if the ants were to stack up independently without building a ring. However, the predicted growth rate based only on these hypotheses is approximately three times too fast to fit the data. Another factor must be coming into play.

We observe that narrow ‘fingers’ of ants that grow upwards from the tower on the rod surface often peel and fall off the rod (see the electronic supplementary material, movie S3). In particular, the fingers that grow above an incomplete ring tend to peel off. We hypothesize that a ring is not stable until it is complete. A partial ring of ants would have to depend only on the adhesive force of a single ant to the central rod. We measure the adhesive force of a single ant to the rod to be 2 ant body weight (2 dynes), which is equivalent to an adhesive pressure of 66 dyne cm^−2^ acting on an ant area of 0.03 cm^2^. The attachment strength mean and standard deviation for all trials (*N*=25) were 2.56 and 1.04 dynes, respectively. The decrease in attachment strength with increasing rod diameter was notable: the means were 3.68 (*σ*=1.23), 2.94 (*σ*=0.65) and 1.70 (*σ*=0.63) dynes, respectively, for rods of diameter 4.7, 12.8 and 14.5 mm.

To show that ants in a ring adhere to the rod more strongly than a single ant to the rod, we apply Laplace’s Law, *P*=2*T*/*Dh*, where *P* is pressure on the central rod, *T* is tensile force between ants and *D* is the rod diameter. On rod diameters ranging from 4 to 19.5 mm, the adhesive pressure *P* ranges from 4500 to 920 dyne cm^−2^, which is 70 to 14 times stronger than the adhesive pressure of a single ant. Ants that build on rod diameters wider than 270 mm do not benefit from forming a ring. However, for the range of rod diameters in our study, the formation of a ring clearly increases the stability of the tower.

The growth rate is governed by the time until the last space in a layer is filled. In §3, we derive the following formula for the expected time for a new layer to form, based on maxima of independent exponential distributions,

4.1 $$E[T]=\sum_{k=0}^{k=S}\frac{\lambda }{S-(k-1)}\approx \lambda \log(S),$$

where *S* is the number of spaces in the ring. To test formula (4.1) of our ring-fill model, note that it predicts different rates for different diameters *D*, since *S*=*πD*/*w*. Prior to deriving (4.1), our trials used a single rod diameter. We ran a large set of trials on rod diameters ranging from 4 to 19.5 mm. The time to complete a ring, shown in figure 5*d*, is a good fit with an *R*^2^ of 0.66 until reaching a rod diameter of 14 mm.

**Figure 6.**
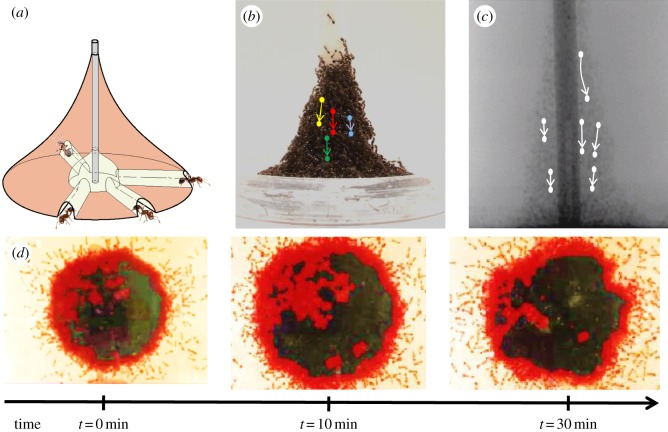
Flowing and rebuilding of ant towers. (*a*) Schematic of the tunnels observed underneath the ant tower. (*b*,*c*) Side views by video camera and X-ray showing ants sinking within the tower. The solid circles represent the characteristic trajectories of ants tracked over a period of 15 min. (*d*) View of the tower from below. The colours indicate the motion of the ants, black referring to no motion and red indicating the flow of traffic. The tunnels migrate, merge and disappear with time.

After about 10 min, the tower begins to sink even as it continues to be built. Ants on the surface (exterior) of the tower move rapidly in all directions atop the other ants, as shown in the electronic supplementary material, video S1. They tend to maintain their relative positions as the entire mass of ants sinks and exits through tunnels on the bottom, as shown in figure 6*a*. At high speed, the surface movements are a blur through which the sinking interior ants are visible. Through this top layer of ants, we could readily see interior ants slowly moving downwards. The sinking rate is approximately $0.38\pm 0.21\;\mathrm{mm}\;\min^{-1}$ based on 10 ant tracks from two towers (examples of four tracks are given in figure 6*b*). From the high-speed photography, the estimated sinking rate was $0.4\pm 0.2\;\mathrm{mm}\;\min^{-1}$ based on 15 ant tracks from three towers.

X-ray spectroscopy of fire ants that have ingested a radiographic contrast medium confirms the tower sinking (see the electronic supplementary material, movie S4). Figure 6*c* shows the downward trajectories of six ants. The iodine drink and X-ray tomography techniques were newly developed for this research, and may be useful in other studies of ant and other animal movement not visible to the eye.

Where do the sinking ants go? Figure 6*d* is a time sequence viewed from below the transparent Petri dishes. We know that ants move downward until they reach the tower base. As depicted in figure 6*a*, they exit through tunnels on the bottom. The fire ants build their tunnels along the base surface, extending to the outskirts of the tower (see the electronic supplementary material, movie S5). Ants enter the tunnels from inside the tower and exit, thereby joining the set of ants that move rapidly on the tower surface. Ants thus appear to clear a path in the tower similarly to removing soil underground. We never observed horizontal movement above the base. We note that ants do not remove other ants from inside the tower. Rather, they push other ants away in order to make a tunnel.

When an ant exits the base of the tower, it joins the other ants that move on the tower surface. We observe no change in the behaviour of the ants on the surface after the tower reaches its maximum height. The growth process continues just as before, including the growth of ‘fingers’ that peel off the rod. Hence, the bell shape does not change.

## 5. Discussion

In the beginning of this study, we asked how fire ants could build such tall towers without being crushed at the bottom of the tower. The ant’s solution is to construct a tower of constant strength. This tower is governed by a single parameter, the strength of an ant, which we found to be three ants. This ant strength was shown to be constant across both building times and different rod diameters. Such a result is consistent with the concepts of decentralized control, as an individual ant’s behaviour is not influenced either by macroscopic features or by its location with respect to the macroscopic structure. For example, an ant in the middle of a tower should not behave differently if below it there are four levels or 14, because its local information would be the same in both cases. The perpetual growth process at the tower top, too, is consistent with decentralized control, because the shape—the local information available to ants on the surface—does not change.

### 5.1. Ant strength influences tower shape

The measurement of this ant strength in our work may have significance in predicting the limits of fire ant architecture. This ant strength can also be construed as the maximum force an ant will sustain before it lets go and departs its position in the tower. In other words, this is the yield stress of a fire ant, the stress at which an ant transitions from being solid to fluid. In tests with a rheometer, across a range of shear rates, we find aggregations of fire ants also apply constant shear stresses to each other of about 2 body weights before releasing their grip. Physically, this results in the ants appearing as shear thinning: they have viscosities that decrease with shear rate [15]. We speculate that this range of two to three body weights allows the ants to protect themselves from injury when in large crowds.

The value *α*=3.0 is larger than the value 1.5 reported as the weight borne by fire ants at the bottom layer of a raft [17]. We speculate that the difference in values is due to the difference in the growth processes. The ant raft starts out thick and flattens as ants attach to the perimeter. The limiting load of 1.5 is minimal, in the sense that a larger diameter but thinner raft would not have enough active ants on the top level to keep ants below them stationary. By contrast, the tower starts out short and grows taller as ants attach to the column and surface. The limiting load of 3.0 is maximal, in the sense that a larger load would induce ants below to exit the tower.

In this work, we hypothesize that the fire ants build a tower without ‘knowing’ they are doing so. According to our hypothesis, the tower results from fire ants following the same set of rules that we previously used to accurately predict the shape and growth rate of a different structure, the ant raft [17,18], formed when the ants are in water. The ants may simply be moving randomly atop other ants until they occupy an empty space adjacent to a stationary ant. In the case of the tower, the only apparently visible empty spaces are around the central rod immediately above the current top of the tower. Because each ant only sustains a maximum amount of force, unstable portions collapse, leading to a tower of constant strength as a stable structure. Because an ant’s adhesion to the rod is much weaker than the radial force resulting from a complete ring of ants gripping each other, the growth rate is limited by the time to complete a ring. This predicted growth rate is confirmed by experiments with different rod diameters. To the best of our knowledge, this is the first example of two very different structures being built by a swarm, where individuals follow the same simple rules, and the difference arises from the environment and mechanical properties.

Future work is needed to determine whether these same rules suffice to explain the building of other fire ant structures, such as bridges used to cross chasms. Work is also needed to link these proposed rules to those found for traffic flows in ants, or to swarm behaviours in other ant species and in organisms such as fish and bees [2,3,25–27]. Research by Cassill *et al.* suggests heterogeneity as another line of future work. They find that during raft-building, small workers behave differently from big workers and matriarchs [28]. We did not track ants by size. Different-sized ants might contribute differently to the building speed and stability of the towers.

The perpetual sinking and rebuilding of the tower also raises a number of questions. Fire ants are clearly wasting energy to constantly rebuild the tower. We speculate that this energy use may be rationalized by the potential benefits from rebuilding the tower, which we outline here. The tunnel building at the base of the tower creates pathways to transfer brood inside the tower, where they are protected. The tower sinking into the tunnels may arise as a consequence of this transport process. Rebuilding the tower helps to clean and remove debris from the outside surface of the tower. Rebuilding the tower may permit ants within the tower to rest on an hourly basis before they support the weight of the tower again. Rebuilding also enables the removal of high stress concentrations, which would naturally arise from deviations from the ideal bell shape. In any case, the perpetual recirculation indicates that the tower itself is very much a living structure, much like its constituents, the ants.

The fire ant raft, once built, is static rather than dynamic. The ants do not waste energy to circulate through the raft or to constantly rebuild it [17]. This contrast with the tower suggests an altogether different explanation for the tower’s perpetual sinking and rebuilding. Perhaps, the tower is an epi-phenomenon from an evolutionary perspective. We speculate that the selection pressure for successful rafts is much greater than the pressure for successful bivouacs. The former are necessary to keep the colony alive for about a month every year. The latter are helpful for only a few days each year, and may not be necessary for survival. Since the same individual behaviours lead to both structures, it is possible that the tower arises as an ancillary consequence of the selection pressure for raft construction, rather than as a behavioural phenomenon in its own right.

### 5.2. Why is the ring the bottleneck?

Why is not the geometrically increasing number of ants needed for the tower height to increase from *n* to *n*+1 the bottleneck limiting the tower growth rate, rather than the constant number needed for the next ring? To answer this question, we observe the tower surface and the ant movements on the surface more carefully by slowing the video replay. In §3, we combine these observations with a mathematical estimate of the ratio of tower surface area to tower base area to explain why ring formation is the bottleneck of the growth rate.

Although the only easily visible empty spaces were those above the top tower ring, our closer observations revealed that the tower surface was not smooth. Ants walking on this uneven surface would encounter a stationary ant, one with another ant atop it. The walking ant would then be trapped into being stationary, just as it would fill an open ring slot. In this fashion, the entire surface of the tower was constantly thickening. The tower did not grow level *n*+1 by keeping levels 1 to *n* fixed, and adding ants below level *n*. Instead, the tower constantly thickened as ants attached over its entire bumpy surface. According to our hypotheses, the time for an opening adjacent to a bump to be filled must have been similar to the time for a ring slot to be filled, since the ants would have been making the same kind of random movements. The difference in the two processes, ring filling and thickening, was that new bumps were constantly being created. Thickening did not have to slow down due to a decrease in the number of places where thickening could occur. The rate of tower thickening, in number of ants per unit time, was therefore proportional to the tower surface area.

The remainder of the analysis depends on a mathematical comparison of the tower surface area to the tower base area. Here is a simple proof that the tower surface area is greater than the area of the tower base: remove all ants from the tower except those on the surface; shine an imaginary light downwards on the tower; the ant shadows cover the base and have greater density than the ants that were there. In §3.3, we compute by calculus a more accurate estimate the actual surface area, approximately (1+1/*α*)^3^ times the area of the base. By equation (4.1), the number of ants that must be added to the tower to increase the height from *n* to *n*+1 is (1+1/*α*) times the area of the base. The surface has (1+1/*α*)^2^≈2 times the number of ants that must be added to the tower to grow it one level. Therefore, tower thickening adds new ants at a rate that keeps up with the number of ants needed to grow the tower. No matter how large the base or diameter, the time for enough ants to join the tower to support another layer remains constant. This completes the explanation of why ring formation is the bottleneck.

## 6. Conclusion

Our study demonstrates how fire ants self-assemble to build towers as temporary shelters. The bell-shaped towers evenly distribute stress inside the tower so that each ant is only supporting three ants. The ants do not attempt to build this shape, but rather it emerges from a process of trial and error, with the most stable shape remaining standing. We also use modelling to predict the speed of tower construction. Our model relies upon a single experimentally measured value, the time it takes an ant to fill an empty slot on the tower. Upon observing the towers for very long times, we observe that towers also undergo continual decay, possibly due to yield stress, and reconstruction. This work may lead to further understanding of large-scale structures built by organisms and robots.

## Supplementary Material

Shape Data AntTower

## Supplementary Material

Slot Time distribution AntTower

## Data accessibility

The datasets supporting this article have been uploaded as part of the electronic supplementary material.
